# Notes of Professor Gaston’s Lecture on Malignant Tumors

**Published:** 1887-05

**Authors:** J. E. Kintz

**Affiliations:** Somerset, Ohio


### Southern Medical Record.

A MONTHLY JOURNAL OF PRACTICAL MEDICINE.

☞All Communications must be addressed to R. C. WORD, M. D., Managing Edito

"*Quicquid Præcipies Esto Brevis.*"

VOL. XVII. ATLANTA, GA., MAY, 1887. No. 5.

### ORIGINAL AND SELECTED ARTICLES.

### NOTES OF PROFESSOR GASTON'S LECTURE ON MALIGNANT TUMORS.

BY J. E. KINTZ, M. D., OF SOMERSET, OHIO.

Under the term "malignant" are comprised those morbid products which have, within a variable period of time after their formation, the effect not only of destroying the tissues in which they are found but also the life of the patient. They are usually described under the name of heterologous, heteroclite or heteromorphous products in reference to their want of similarity to the natural structure of the tissue in which they are found, but the term malignant applies to all on account of their destructive disposition.

The formations under this head are scirrhus, encephaloid, colloid, melanosis, and cancroid. Tubercle is not usually considered under this classification by surgeons.

It would seem an unfortunate circumstance that the term cancer has been applied to this form of morbid growth, as its application is entirely figurative and calculated to mislead the mind of the student. The prevalence of cancerous disease is very great, as shown by the statistics taken in England a number of years ago. By the above statistics we find that by far the greatest number occur in females, the ratio being about three females to one male. No reason can be assigned for that difference other than the frequency of the disease in the mamma and uterus.

These various forms of morbid growth, although differing very widely in many of their characteristics, possess some features in common, which we will consider before speaking of each separately:

1. They are all deficient in plastic material, but contain a large amount of albuminous substance, or the protein principles of the blood.

2. They are all found, under the microscope, to consist of two parts—a fibrous stroma, and granules or cells, which seem to bear the relation of contained and containing parts. The cells often contain nuclei and nucleoli.

3. They occur in nearly all the organs and tissues of the body, in both sexes, and at all periods of life, in all temperaments, in persons of all occupations, often existing at the same time in a number of localities. They frequently display a very marked hereditary tendency.

4. They present themselves under different forms; generally as a circumscribed tumor, but frequently as a stratum or an infiltration.

5. They are all deposited as a secretion in fluid form, but soon become of a concrete character. After a time they work their way to the surface by exciting ulcerative action in the surrounding tissues. The sore which results is incapable of forming healthy granulation.

6. They are all of constitutional origin, or connected with a contaminated condition of the blood and solids. They usually make their appearance without any assignable cause, but may sometimes appear as the result of an injury. They always manifest a tendency to return after extirpation, either at the original site or at some new point. Their progress is usually rapid, causing death in from nine to eighteen months after their first appearance. As they advance they are apt to involve the neighboring lymphatic ganglions.

The cancer cells are considered by the majority of pathologists to be of great diagostic value. In shape, the cancer cells are generally spherical, and about one-thousandth of an inch in diameter, have oval eccentric nucleus, and one or more nucleoli; as a rule, the largest and most perfect cells are found in scirrhus, particularly those of slow growth. The less perfect are seen in encepaloid and all rapidly developiug forms of the disease.

### SCIRRHUS.

Scirrhus is a term employed to designate a morbid product, which is denser and firmer than any of the natural tissues, except bone, cartilage and tendon. It is often called "hard cancer." Scirrhus is one of the most frequent occurring of all the malignant growths. It occurs at various periods of life, but rarely till after middle age. Scirrhus of the skin and liver sometimes occurs at a comparatively early period ; but elsewhere it does not often occur until after the age of forty. Females are much more liable to scirrhus than males. Certain organs and tissues are more prone to scirrhus than others. The liver, mamma, and uterus are very liable to suffer, while the testicle is very seldom or never attacked. The stomach, anus, rectum, and colon are often involved in both sexes ; of these the rectum seems to suffer most frequently. The bones, muscles, tendons, ligaments, aponeurosis, vessels, nerves, salivary glands, brain, lungs, spleen, and urinary organs are almost entirely exempt from this form of morbid growth.

Scirrhus may appear under several varieties of form, as the tuberoid, stratiform, and infiltrated—the first of which is of the greatest importance surgically, as the other two occur principally in the internal organs. The best example of the tuberoid variety occurs in the mammary gland. The tumor consists, sometimes, of a simple mass, but generally it is made up of several which, together, may form a growth as large as an orange, or a man's fist. It is hard, dense and inelastic, and almost incompressible ; when cut, it produces a peculiar grating sound. The tissues, sometimes, afford the tumor an imperfect capsule, but usually there is no such investment, the heteroclite matter being often spread in a very irregular manner, somewhat resembling the claws of an animal.

### STRUCTURE.

A section of scirrhus in its mature stages presents a whitish, homogenous appearance, and, upon being scraped, it affords a peculiar lactescent fluid called cancer-juice. Scirrhus has a very feeble circulation, its vessels being very small and, probably, wholly derived from the surrounding parts. No nerves have been traced into its substance, but their presence is inferred from the sharp, lancinating pain which is so characteristic of the disease. Lymphatics also exist, which is proved by the fact that if arsenic be applied to an open scirrhus it is often absorbed, producing the same effect as when taken into the stomach.

### CHEMICALLY.

Scirrhus is found to consist largely of albumen and fatty matter, the two forming more than half the entire mass. Earthy salts are found in considerable quantities, the greater part being in the form of sub-phosphate of lime. The minute structure of scirrhus consists of two parts, a fibrous network and soft granular matter. The essential elements of scirrhus consists, mainly, of cells and free nuclei, lying in a transparent matrix. The cells are of various shapes; in size they range from the one-sixhundreth to the one-seventeen-hundreth of an inch in diameter; they have each a distinct wall, and generally inclose one or more nuclei.

### PROGRESS.

Scirrhus generally progresses more slowly than the other heteromorphus growths. It seldom becomes an open ulcer under twelve, fifteen or eighteen months. The color is always dark-purple or livid, the vessels immediately concerned in its production being enlarged and deeply congested. When it breaks down, an unsightly ulcer is exposed, having hard, steep and rounded edges, and a foul-looking bottom, generally incrusted with spoiled lymph. The discharge is always of a sanious, ichorous or sanguinolent nature, more or less fetid and irritating. It tarnishes silver, and imparts a green color to syrup of violets, and on mixing with sulphuric acid evolves a peculiar gas similar to sulphuretted hydrogen. No granulations ever form upon an ulcer. The tendency is to spread and to contaminate the surrounding structures. The structures most likely to suffer in this way are lymphatics.

### SYMPTOMS

Are local and constitutional. In taking hold of such a tumor, we find it very hard and dense. It feels firm, incompressible and inelastic. At first it is circumscribed, but later it contracts adhesions to the surrounding parts and becomes firmly fixed in its position. The pain in scirrhus is sharp, darting and lancinating, causing a sensation as if needles were thrust into it. In the earlier stages it produces very little constitutional disturbance, but after the tumor has gone on for some time sleep is interrupted by the pain; the secretions are disturbed, bowels are irregular, and occasional attacks of fever; the countenance becomes sallow; sometimes there is diarrhœa. The patient dies completely exhausted. During the progress of the disease, scirrhus deposits often appear in other parts of the body.

### ENCEPHALOID.

Encephaloid has various synonyms, the most common of which are soft cancer, medullary sarcoma, cerebriform cancer, and fungus hematodes. The disease is more common in the mamma, eye, testicle, uterus, liver, lymphatic ganglions, and bones than elsewhere. It seems to occur more frequently in males than in females. Chemical composition of encephaloid shows a close resemblance to that of scirrhus. The encephaloid matter presents itself under three forms, as a tumor, a stratum, and an infiltration, the first being the most common and of the greatest importance surgically. It varies in size from that of a pea to an adult head ; its shape is generally somewhat ovoidal, and its surface more or less lobulated. When seated in an external structure it has generally a well-marked cyst, of a grayish or whitish appearance. Extending from the inner surface of this envelope are numerous processes passing through the interior of the diseased mass, intersecting each other and forming cells or cavities for the accommodation of new matter. The encepaloid matter, freed from its stroma, is of a soft, jelly-like consistence, resembling somewhat the fœtal brain, of whitish or slightly reddish tint. It is coagulable by heat, alcohol and the acids. If a section of encephaloid be made it presents quite a density of appearance and consistence. One part will be found soft and white, like brain ; another feel and look and cut like fibro-cartilege, while a third may be composed of a reddish, semi-concrete substance. To the latter the name of hematoid has sometimes been applied as expressive of its blood-like structure. It looks more like firmly coagulated blood than any other structure to which it can be compared. Sometimes a more solid substance is mixed up with the hematoid, as fibrous or fibro-cartilaginous structure. When this is the case it creaks under the knife, and cuts somewhat like raw turnip or bacon-rind. This form seems to occur most commonly in the mamma, liver, testicle and lymphatic ganglions. Encephaloid sometimes occur as an infiltration in the liver, lungs, uterus, and lymphatic ganglions. This variety is not common. The stratiform variety occurs, sometimes, but rarely, in the cellular tissue of the stomach and rectum, and beneath the pleura and peritoneum. The encephaloid is a highly vascular structure and has a very high degree of vitality, growing very rapidly, and often attaining an enormous size in a few months after its first appearance. It seems to have a double circulation—one peculiar to itself, the other common to it and the tissues in which it is found. The walls of the vessels are very brittle, and often give way, giving rise to dangerous hemorrhage. It is thought that both nerves and absorbant vessels exist in this form of morbid growth. Serous cysts are sometimes found in encephaloid tumors. When encephaloid starts it usually goes steadily onward till it destroys life. The time at which this takes place varies ; on an average, it is from nine to twelve months. Death sometimes occurs much earlier, and may be deferred much later, in exceptional cases. The period at which encephaloid becomes an open ulcer is indefinite ; but when it does occur it is always characteristic. Its edges are thin and undermined, jagged or irregular, and its bottom has a foul, bloody-fungus appearance. The discharge is usually profuse, and of a sanious, bloody or gangrenolent character. A development of laudable pus is never seen. Copious hemorrhage often takes place, particularly in the hematoid variety, and rapidly undermines the health. Lymphatic enlargement usually occurs at an early period of the disease. Constitutional cachexy is always well marked in the advanced stages of the disease. Encephaloid is always distinguished by its comparative softness, its great bulk, and its lobulated surface. The pain is generally slight at first. There is nearly always enlargement of the subcutaneous veins. In its earlier stages the tumor is movable, but in the advanced stages adhesions are formed, and it becomes permanently fixed.

[To be concluded in our next.]

